# Severity of Lesions Involving the Cortical Cholinergic Pathways May Be Associated With Cognitive Impairment in Subacute Ischemic Stroke

**DOI:** 10.3389/fneur.2021.606897

**Published:** 2021-06-08

**Authors:** Huo-Hua Zhong, Jian-feng Qu, Wei-Min Xiao, Yang-kun Chen, Yong-lin Liu, Zhi-qiang Wu, Dong-hai Qiu, Wen-cong Liang

**Affiliations:** ^1^Department of Neurology, Dongguan People's Hospital, Dongguan, China; ^2^Graduate School, Guangdong Medical University, Dongguan, China

**Keywords:** stroke, cortical cholinergic pathways, brain MRI, cognitive impairment, white matter lesions

## Abstract

**Purpose:** Impairment of cortical cholinergic pathways (CCP) is an important risk factor for chronic vascular cognitive impairment. However, this phenomenon has rarely been studied in post-stroke cognitive impairment (PSCI). We investigated the relationship between PSCI and CCP lesions assessed by structural magnetic resonance imaging (MRI).

**Patients and methods:** We prospectively enrolled 103 patients within 7 days of ischemic stroke onset. CCP was measured by the cholinergic pathways hyperintensities scale (CHIPS), which semiquantitatively grades MR lesions strategically located on the CCP identified in human brains. We also measured other MRI parameters, including the location and volumes of acute infarcts, cerebral microbleeds, medial temporal lobe atrophy, and white matter lesions. Neuropsychological assessments were performed using the 60-min modified vascular dementia battery (VDB) at 3 months after the index stroke, and PSCI was defined according to VDB as well as ADL.

**Results:** Of all 103 patients, 69 men (67.0%) and 34 women (33.0%) with a mean age of 57.22 ± 12.95 years, 55 patients (53.4%) were judged to have PSCI at 3 months, including 43 (41.7%) patients with PSCI-no dementia and 12 (11.7%) patients with poststroke dementia. According to the VBD assessment, the most commonly impaired cognitive domain was visuomotor speed (27.2%) followed by verbal memory (25.2%). Univariate analysis showed that patients with PSCI were older; had higher informant questionnaire on cognitive decline in the elderly (IQCODE) scores; had more frequent previous stroke history and atrial fibrillation; and had higher CHIPS scores, more severe white matter lesions, and medial temporal lobe atrophy. PSCI patients also had higher depression scores at 3 months. In the multivariate regression analysis, age, IQCODE score, CHIPS score, and Hamilton depression rating scale score were independent predictors of PSCI. Ordinal regression analysis for risk factors of poor functional outcomes revealed that IQCODE scores and cognitive function status were related to mRS score at 3 months after stroke.

**Conclusion:** In patients with early subacute ischemic stroke, the severity of lesions involving the CCP may be associated with cognitive impairment at 3 months.

**Clinical Trial Registration:** Chinese Clinical Trial Registry, identifier: ChiCTR1800014982.

## Introduction

Poststroke cognitive impairment (PSCI), which includes PSCI-no dementia (PSCI-ND) and poststroke dementia (PSD), is one of the most common outcomes of acute ischemic stroke, occurring in ≥80% of survivors (1, 2). It has a negative effect on functional outcomes (1, 3) and predicts the recurrence of vascular events (4). However, the precise pathological mechanisms of PSCI remain unclear.

Acetylcholine (ACh) functions as an excitatory neuromodulator of the cerebral cortex and participates in nearly all aspects of cortical function, and its reduction may be closely related to the occurrence of PSCI (5). ACh is mainly located in the axons of cholinergic neurons, which is the synthesis of choline and acetyl-CoA in one step by choline acetyltransferase (ChAT). ACh depends on the vesicle ACh transporter (VAChT) to be transported and stored in synaptic vesicles. During an action potential, under the mediation of Ca^2+^, ACh vesicles fuse with the presynaptic membrane, and the vesicles rupture. ACh enters the synaptic cleft and binds to the ACh receptors of the presynaptic and postsynaptic membranes, thereby causing physiological effects. The release of Ach is regulated by many factors. Activation of noradrenergic α1 receptors may increase cortical Ach release, and glutamate can excite basal forebrain cholinergic neurons and increase cortical high-affinity bile alkali uptake and ACh release (6–8). There are two types of ACh receptors: muscarinic acetylcholine receptors (mAChRs) and nicotinic acetylcholine receptors (nAChRs), located in the presynaptic and postsynaptic membranes in the brain, playing important roles in regulating the release of glutamate, γ-aminobutyric acid, dopamine, norepinephrine, and other neurotransmitters related to cognitive function (9, 10). ChAT is the most specific marker for cholinergic neurons. The cortical cholinergic pathway (CCP) was discovered through brain ChAT immunohistochemical staining and magnetic resonance imaging (MRI) overlay analysis technology (11). The CCP is one of the major cholinergic structural connections in the brain. It includes the diagonal band of Broca, the medial septal nuclei, and the nucleus basalis of Meynert (nbM) and projecting fibers from these cells (12). Severe neurofibrillary degeneration of cholinergic cell bodies in the nucleus basalis and correspondingly severe denervation of neocortical cholinergic projections was reported in a patient with Alzheimer's disease (AD) (13). Damage to cholinergic fibers and a decrease in cholinergic neurotransmitters may be involved in cognitive impairment.

The CCP represents fibers that project from the nbM to the cortex and amygdala and also branch into the medial and lateral pathways (11, 14, 15). In 2005, Bocti et al. developed the cholinergic pathways hyperintensities scale (CHIPS) based on immunohistochemical research to measure CCP (16). Lim et al. first established a case-control study of patients with acute ischemic stroke, using a modified version of CHIPS to evaluate the MRI data and reported that disruption of the cholinergic pathways and major hubs of large-scale neural networks might contribute to newly developed dementia after acute ischemic stroke (17). However, the sample in this previous study was relatively small, and the potential relationship between CCP involvement and PSCI in patients with acute or early subacute ischemic stroke was not studied in detail. Hence, we conducted the present study to explore the relationship between the degree of CCP involvement and PSCI in patients with early subacute ischemic stroke.

## Methods

### Patients

The study was conducted in Division I, Department of Neurology, Dongguan People's Hospital (Dongguan, China) between September 1, 2018, and July 31, 2019. The inclusion criteria for the study were (1) age >18 years, (2) first or recurrent early subacute ischemic stroke occurring within 7 days before admission (the ischemic stroke diagnosis was made in accordance the American Heart Association Stroke Council criteria) (18), and (3) that a complete brain MRI examination had been performed. The exclusion criteria were (1) transient ischemic attack, cerebral hemorrhage, epidural or subdural hematoma, or subarachnoid hemorrhage; (2) prestroke cognitive impairment, which was assessed using the informant questionnaire on cognitive decline in the elderly (IQCODE) in hospital (19); (3) coexisting conditions (beside stroke) that affect cognitive function (e.g., major depression or anxiety, chronic alcoholism, previously diagnosed psychiatric conditions, epilepsy, severe traumatic brain injury, Parkinson's disease, multiple sclerosis); (4) lack of complete clinical or MRI data; (5) death during hospitalization; (6) severe stroke indicated by a National Institutes of Health stroke scale (NIHSS) score ≥15 points at discharge; (7) patients with severe comorbidities (e.g., liver, kidney, heart, or respiratory failure or malignant tumors); (8) severe hearing disabilities, sight disabilities, or language disorders at discharge according to physical examination; and (9) refusal by patients or their relatives to provide informed consent.

### Collection of Demographic Data

Demographic and clinical variables (i.e., age, sex, years of education, onset time, vascular risk factors, history of stroke, and neurological deficit status assessed using the NIHSS) were collected. Preexisting cognitive status was evaluated using the IQCODE within 3 days after admission. The IQCODE questionnaire consists of 16 items that assess impairment in the patient over the previous 10 years. The IQCODE score ranges from 16 (marked improvement in all items) to 80 (marked worsening in all items). A total score >51 points is considered to indicate a preexisting cognitive impairment (19).

### MRI Assessments

Brain MRI scanning, including T1-, T2-, diffusion-, and susceptibility-weighted imaging sequences were performed on each participant using a 3.0-T system (Sonata; Siemens Medical, Erlangen, Germany) within 7 days after admission. Diffusion-weighted image spin-echo echo-planar imaging (repetition time [TR]/echo time [TE]/excitation: 2,162/76/1; matrix: 128×128; field of view (FOV): 230 mm; slice thickness/gap: 6 mm/1 mm; echo-planar imaging factor: 47; acquisition time: 25.9 s) with three orthogonally applied gradients was used with a *b* value of 0 and 1,000. Axial spin echo T1 (TR/TE/excitation: 488/15/1; FOV: 230 mm, slice thickness/gap: 6 mm/1 mm; matrix: 256 × 256; time of acquisition: 1 min 24.8 s) and turbo spin echo T2 (TR/TE/excitation: 3,992/110/2; turbo factor: 15; FOV: 230 mm; slice thickness/gap: 6 mm/1 mm; matrix: 512 × 512, time of acquisition: 1 min 55.8 s) images were also captured.

**Figure 1:**
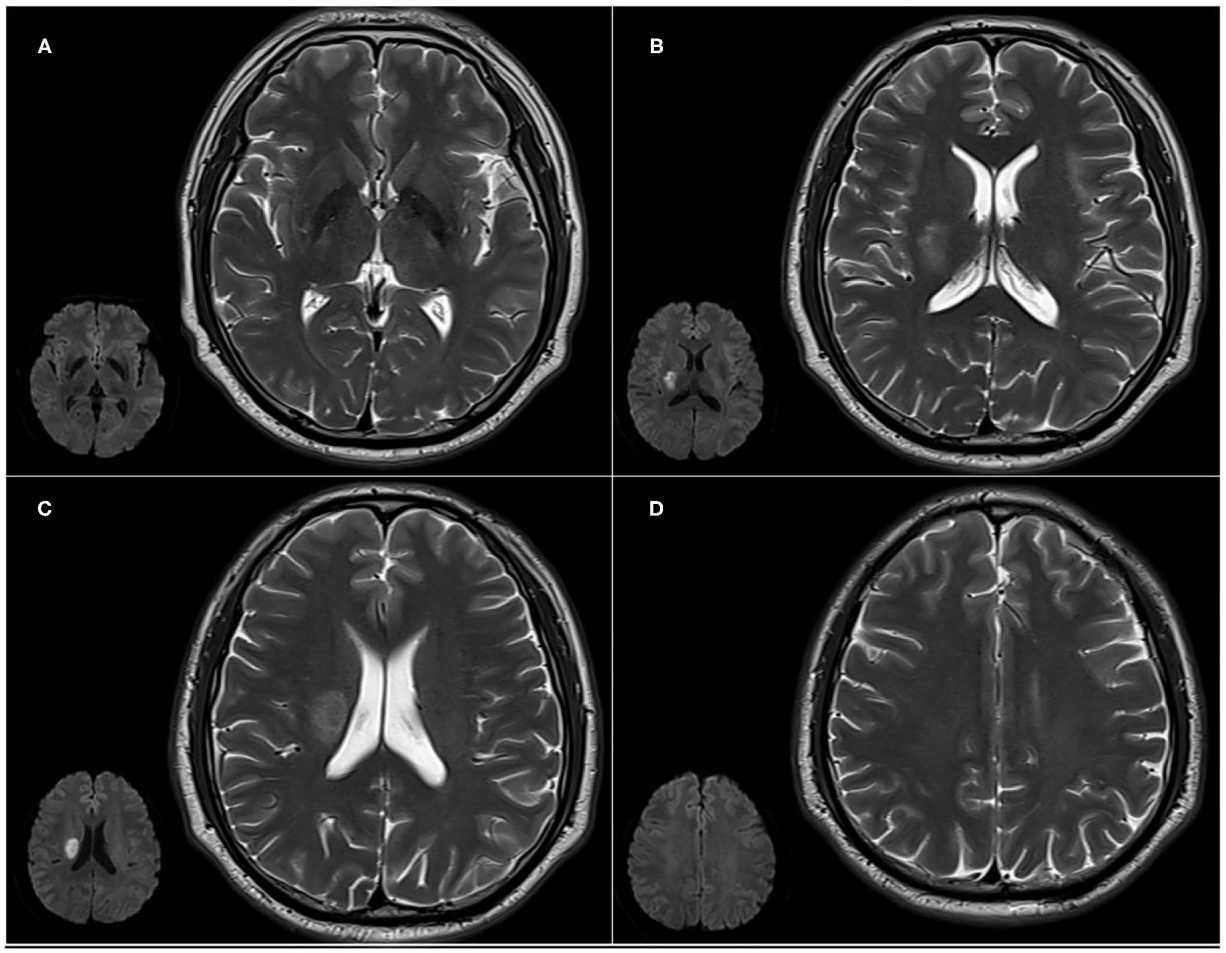
Cholinergic Pathways Hyperintensities Scale (CHIPS) scores from representative patients with both early subacute ischemic stroke and white matter lesions (WMLs). Slices: **(A)** low external capsule layer (bilateral posterior zones score are 1 each, bilateral anterior zones score 0); **(B)** high external capsule layer (bilateral posterior zones score 1 each, bilateral anterior and cingulate gyrus zones score 0); **(C)** corona radiata (right posterior zone score 1, bilateral anterior and cingulate gyrus zones score 0); and **(D)** the semioval center (bilateral anterior and posterior zones score 0).

A neurologist (JFQ) measured the MRI variables as follows:

***(1) Assessment of lesions involving the CCP***. Lesions involving the CCP were assessed using CHIPS (16), which was developed based on immunohistochemical tracings of the cholinergic pathways in humans, superimposed onto a structural MRI image (11). Measurement zones within four index slices of white matter regions were demarcated on axial T2-weighted images of the lateral ventricles and the third ventricle. Slices: (A) low external capsule layer (four separate zones: bilateral anterior and posterior zones), (B) high external capsule layer (six separate zones: bilateral anterior, posterior, and cingulate gyrus zones), (C) corona radiata (six separate zones: bilateral anterior, posterior, and cingulate gyrus zones), and (D) semioval center (four separate zones: bilateral anterior and posterior zones). The severity of white matter hyperintensities (WMHs) in each zone was scored as (1) normal (0 points), (2) <50% of the region involved (1 point), and (3) ≥50% of the region involved (2 points). Severity scores were weighted according to the distribution density of cholinergic fibers. The weighting coefficients were as follows: 4 for slice A, 3 for slice B, 2 for slice C, and 1 for slice D. The score for each region was obtained by adding all points from the measurements of small zones and multiplying them by the weighting coefficient. The maximum score per hemisphere was 50, and the total maximum score per scan when combining scores for all four regions was 100. The CHIPS total score showed a good correlation with lesion volume within the cholinergic pathways (Spearman's = 0.87, *p* < 0.0001). Higher CHIPS scores suggest more severe CCP impairment (16). Lesions involving the CCP included acute infarction, old infarction, or WMHs (Figure 1) (20).

***(2) Acute infarcts***. Acute infarcts were defined as regions of restricted water diffusion identified on diffusion-weighted imaging with *b* values of 1,000 together with hypointensity on the corresponding apparent diffusion coefficient map. The sites of acute infarcts were divided into cortical regions (frontal, temporal, parietal, and occipital lobes), subcortical regions (white matter, basal ganglia, and thalamus), and infratentorial regions (i.e., brainstem and cerebellum). The contours of an acute infarct on diffusion-weighted imaging were manually outlined. The total volume was subsequently calculated by multiplying the total area by the sum of the slice thickness and the gap.

***(3) White matter lesions (WMLs)***. The severity of WMLs, including deep white matter hyperintensities (DWMH) and periventricular hyperintensities (PVH), were scored using fluid-attenuated inversion recovery images with the Fazekas scale (range 0–3) (21).

***(4) Medial temporal lobe atrophy (MTLA)***. MTLA was assessed using the Scheltens' scale (22) in which coronal MRI sections are used to judge the severity of MTLA based on standard images (range from 0 to 4, no atrophy to severe atrophy).

***(5) Cerebral microbleeds***. These were defined as small (2–10 mm) hypointense lesions with a clear margin in susceptibility-weighted imaging. Symmetric basal ganglia calcification and flow void artifacts of the pial blood vessels were excluded.

### Cognitive and Psychological Assessments

Neuropsychological assessments were performed at 3 months through a face-to-face interview with patients after the index stroke by a trained neurologist (HHZ), who was blinded to all clinical data. We did not assess cognitive impairment in the acute phase because, in acute stroke, assessment of cognitive function is not reliable because of cognitive status being unstable, and the severity of disease can preclude lengthy neuropsychological testing (23, 24). We did not assessed the cognitive function used by the Montréal Cognitive Assessment because it is commonly used as a screening tool rather than a diagnostic criteria (25, 26). We used a more comprehensive neuropsychological battery that was a modified version of the vascular dementia battery (VDB) (27, 28), which comprises the following seven cognitive domains: (1) executive functions [frontal assessment battery (29, 30); trail-making test, parts A and B]; (2) attention (digit span: forward and backward) (31), visual memory span (forward and backward) (32) and the auditory detection test (28); (3) language (modified Boston naming test) (33) and verbal fluency (34) (animal and food categories); (4) verbal memory (immediate and delayed word list recall, delayed word list recognition, and immediate and delayed story recall) (35). For this, the tester needs to clearly read 12 related vocabularies or a story to the testee, learn it three times, and then the tester needs to recall the vocabulary or story in real time and then recall the vocabulary or story they just learned 20 min later. During the delayed word list recognition, according to the content just learned, the testee needs to recognize the order of the vocabulary. Next is (5) visual memory (immediate picture recall, delayed picture recall, and delayed picture recognition) (32), visual reproduction I and II and delayed recognition from the Wechsler memory scale III (WMS-III) (32); (6) visuoconstruction [clock drawing test (35), Wechsler adult intelligence scale III [WAIS-III] block design (31) and visual reproduction (copy) from WMS-III (32)]; (7) visuomotor speed [symbol digit modalities test (36), digit cancellation test, and the maze task (28)]. The testee can see the content of the test. In the symbol digit modalities test, he or she needs to simulate different symbols corresponding to different numbers that have been specified, fill in the corresponding numbers in the space below the symbols, and record the number filled in by the tester in 120 s. In the digit cancellation test, the subject needs to cross out the number to be canceled as much as possible within 90 s. The maze task is to calculate the time it takes for the testee to successfully find the exit route of the maze. Apart from the executive domain, impairment of individual cognitive domains was identified if at least 50% of the subtests were below the cutoff points. The cutoff points were derived from a Chinese sample (33) (Supplementary Table 1). The criteria for impairment in any of the seven cognitive components were adjusted for the education level of each patient (28). PSCI refers to a series of syndromes meeting the diagnostic criteria of cognitive impairment within 6 months after an index stroke according to the Diagnostic and Statistical Manual of Mental Disorders (Fifth Edition) criteria (37). We defined PSCI as impairment in at least one cognitive domain; PSCI-ND was defined as impairment in at least one cognitive domain but with normal daily abilities, which were assessed using the Lawton activities of daily living (ADL) scale (38). Cases with simultaneous impairment in at least two cognitive domains and IADL were considered to have PSD. Additionally, we also assessed the anxiety and depression statuses of patients, which were measured using the Hamilton anxiety rating scale (HAMA) and the Hamilton depression rating scale (HAMD), respectively.

### Functional Dependence and Disability Assessment at 3 Months

The functional dependence assessment at 3 months used the ADL scale. The ADL, which includes the instrumental ADL (IADL) and basic ADL (BADL) scales, examines the current functional level of a patient and can identify improvement or deterioration over time. The eight domains of function measured with the IADL scale include the ability to use a telephone, shopping, food preparation, housekeeping, laundry, mode of transportation, responsibility for own medications, and ability to handle finances. The total IADL score is calculated by summing up the points obtained for each item. The maximum IADL score is 32. The BADL scale features six additional questions that measure different levels of toilet activity, feeding, dressing, grooming, and physical activities. The total ADL score is calculated by summing the IADL and BADL scores together. The lowest possible ADL score is 14 points, which indicates that the patient's abilities are completely normal. If the score is >14 points, there is a reduced function, and the highest possible score is 56 points. The severity of disability was measured with the modified Rankin scale (mRS). We defined a poor disability outcome as mRS > 2 points. Death and recurrence of stroke during the follow-up period were also recorded.

### Statistical Analysis

All statistical analyses were conducted using the SPSS Version 24.0 (IBM Corp., Armonk, NY, USA) software. Descriptive data are presented as proportions, means, or medians as appropriate. A univariate analysis was performed to compare putative risk factors between patients with and without PSCI. In the logistic regression analysis, we used a backward elimination procedure. The PSCI served as a dependent variable. Subsequently, risk factors with a value of *P* < 0.05 were analyzed with a multivariate logistic regression analysis using a backward stepwise selection strategy. Correlation analyses were conducted to test collinearity between candidate independent variables. If the correlation coefficient between any of these putative risk factors was ≥0.50, then variables with a smaller *P*-value were entered into the logistic regression. As age and prestroke cognitive status were important risk factors for PSCI, we set up two models, respectively. Model 1 included age, and model 2 included IQCODE score with the rest of the putative factors held constant. The odds ratio of any independent risk factor was interpreted as the risk of non-remission of PSCI, when all other risk factors were held constant. For the analysis of the association between cognitive status and mRS, we constructed an ordinal regression model (dependent variable: mRS grading), adjusted for age education level, IQCODE, NIHSS score at admission, and PVH. The level of statistical significance was set at *P* < 0.05 (two-sided).

## Results

**Figure 2:**
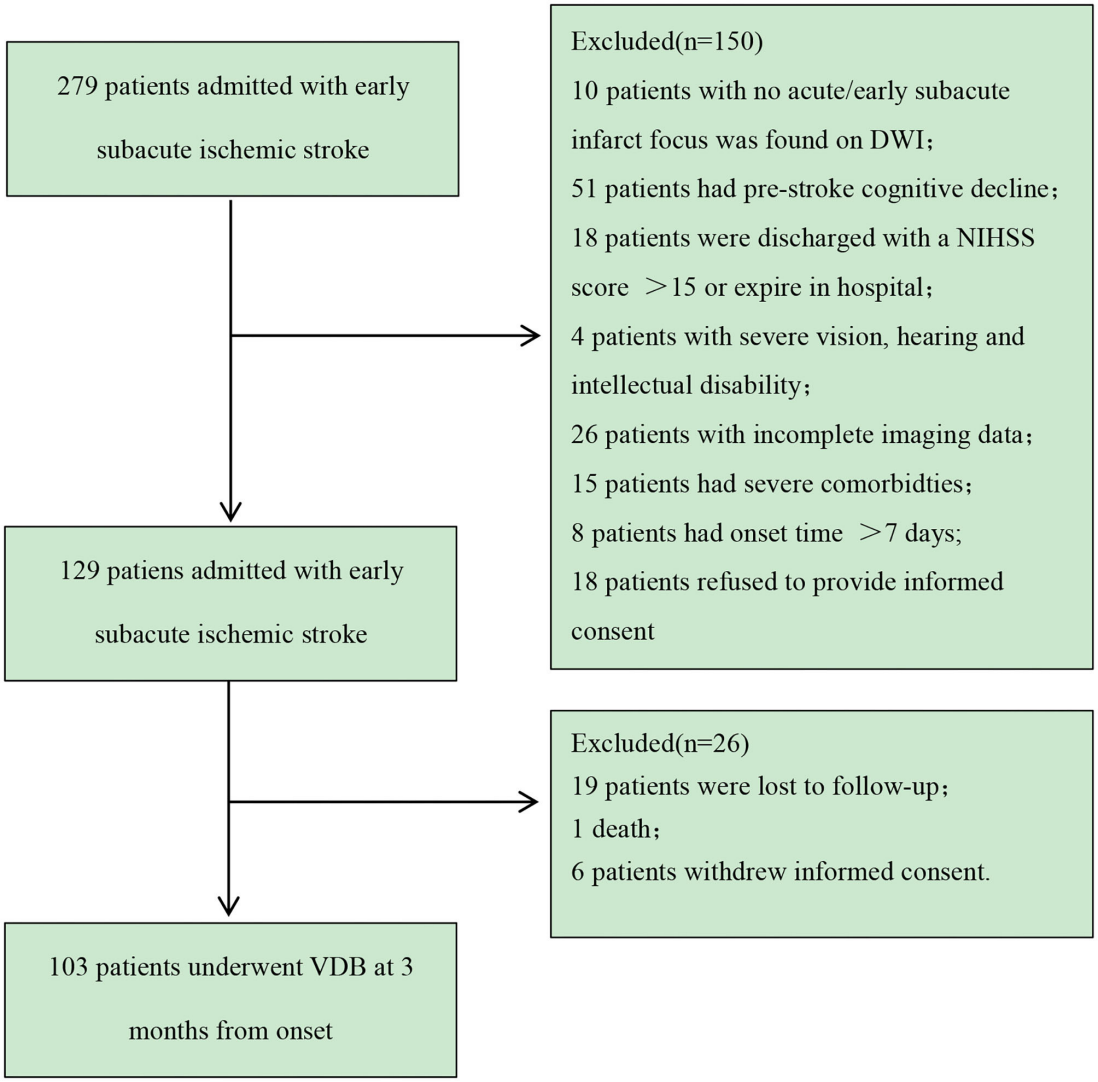
Patient recruitment flowchart. VDB, modified version of the Vascular Dementia Battery; TIA, transient ischemic attack; NIHSS, National Institutes of Health Stroke Scale.

During the study period, 279 Chinese patients were consecutively screened, and 103 patients were included in the analysis. The patient selection process is shown in Figure 2.

Compared with the excluded patients, the study population was younger (57.22 ± 12.95 years vs. 65.74 ± 13.51 years; *P* < 0.001) and had lower NIHSS scores and IQCODE scores (2 [1–4] vs. 4 [2–8.5], *P* = 0.003; 49 [48–49] vs. 50 [48–53.75], *P* < 0.001, respectively); however, there was no significant difference in sex (69 men [67.0%] vs. 117 women [66.5%]; *P* = 0.930), respectively.

The baseline characteristics of the recruited patients are summarized in Table 1. The study sample comprised 69 men (67.0%) and 34 women (33.0%) with a mean age of 57.22 ± 12.95 years. The IADL score is 8 (8–9) and the mRS score was 1 (0–2) at 3 months.

**Table 1:** Comparisons of demographic, clinical, and imaging characteristics between patients with normal cognition and those with PSCI.

	**Whole sample *n* = 103**	**Normal *n* = 48**	**PSCI *n* = 55**	**t/z/χ2**	***P*-value**
Age (years)^a^	57.22 ± 12.95	51.33 ± 13.24	62.36 ± 10.31	−4.667	<0.001
Male^b^	69 (67.0%)	36 (75.0%)	33 (60.0%)	2.608	0.106
Education level^b^				86.878	0.619
Illiteracy (<1 year)	6 (5.8%)	1 (2.1%)	5 (9.1%)		
Primary (1–6 years)	51 (49.5%)	18 (37.5%)	33 (60.0%)		
Secondary (7–12 years)	38 (36.9%)	23 (47.9%)	15 (27.3%)		
University (>12 years)	8 (7.8%)	6 (12.5%)	2 (3.6%)		
IQCODE score^c^	49 (48–49)	48 (48–48)	49 (49–50)	−5.630	<0.001
Smoking history (≥6 months)^b^	37 (35.9%)	19 (51.4%)	18 (48.6%)	0.523	0.469
Hypertension^b^	64 (62.1%)	26 (40.6%)	38 (59.4%)	2.427	0.119
Diabetes mellitus^b^	31 (20.1%)	11 (22.9%)	20 (36.4%)	2.203	0.138
Ischemic stroke history^b^	14 (13.6%)	3 (6.3%)	11 (20.0%)	4.126	0.042
Atrial fibrillation^b^	11 (10.7%)	1 (2.1%)	10 (18.2%)	6.964	0.008
NIHSS score at admission^c^	2 (1–4)	2 (1.5–4)	3 (2–4)	−1.208	0.227
i.v. thrombolysis^b^	16 (15.5%)	7 (14.6%)	9 (16.4%)	0.062	0.803
Stroke subtype^b^				8.343	0.072
Large artery	31 (30.1%)	12 (25.0%)	19 (34.5%)		
Cardioembolism	12 (11.7%)	2 (4.2%)	10 (18.2%)		
Small artery	37 (35.9%)	21 (43.8%)	16 (29.1%)		
Other etiologies	7 (6.8%)	5 (10.4%)	2 (3.6%)		
Unknown etiologies	16 (15.5%)	8 (16.7%)	8 (14.5%)		
**Location of acute infarction**					
Cortical region^b^	47 (45.6%)	18 (37.5%)	29 (52.7%)	2.396	0.122
Subcortical region^b^	72 (69.9%)	31 (64.6%)	41 (74.5%)	1.209	0.272
Infratentorial^b^	29 (28.2%)	18 (37.5%)	11 (20.0%)	3.880	0.049
Infarct volume^c^	1.37 (0.52–8.14)	1.20 (0.38–11.10)	3.74 (0.67–11.93)	−1.690	0.091
Total CHIPS score^c^	16 (4–36)	8 (2.5–18.5)	27 (10–44.5)	−3.611	<0.001
PVH^c^	1 (0–2)	1 (0–1)	2 (1–2)	−2.997	0.003
DWMH^c^	1 (0–1)	0 (0–1)	1 (1–2)	−2.769	0.006
MTLA^c^	1 (0–4)	0 (0–2)	2 (1–4)	−3.596	<0.001
Presence of CMBs^b^	38 (36.9%)	15 (31.3%)	23 (41.8%)	1.130	0.288
HAMA^c^	7 (5–10)	7 (5–10)	9 (5–12.75)	−1.714	0.087
HAMD^c^	8 (5–12)	8 (5–10)	9.5 (6–14)	−2.514	0.012
ADL^c^	14 (14–16)	14 (14–14)	14 (14–19)	−2.802	0.005
IADL^c^	8 (8–9)	8 (8–8)	8 (8–12)	−2.633	0.008
BADL^c^	6 (6–6)	6 (6–6)	6 (6–7)	−3.201	0.001
mRS^c^	1 (0–2)	1 (0–1)	1 (0–2)	−2.075	0.007

a *Mean (SD), t-test*.

b *n (%), chi-squared test*.

c *M(Qu-QL), Mann–Whitney U-test*.

*PSCI, post-stroke cognitive impairment; IQCODE, Informant Questionnaire on Cognitive Decline in the Elderly; NIHSS, National Institutes of Health Stroke Scale; i.v., intravenous; CHIPS, Cholinergic Pathways Hyperintensities Scale; PVH, periventricular hyperintensity; DWMH, deep white matter hyperintensity; MTLA, medial temporal lobe atrophy; CMBs, cerebral microbleeds; HAMA, Hamilton Anxiety Rating Scale; HAMD, Hamilton Depression Rating Scale; ADL, Lawton Activities of Daily Living; IADL, Instrumental Activities of Daily Living; BADL, Basic Activities of Daily Living; mRS, modified Rankin Scale*.

**Figure 3:**
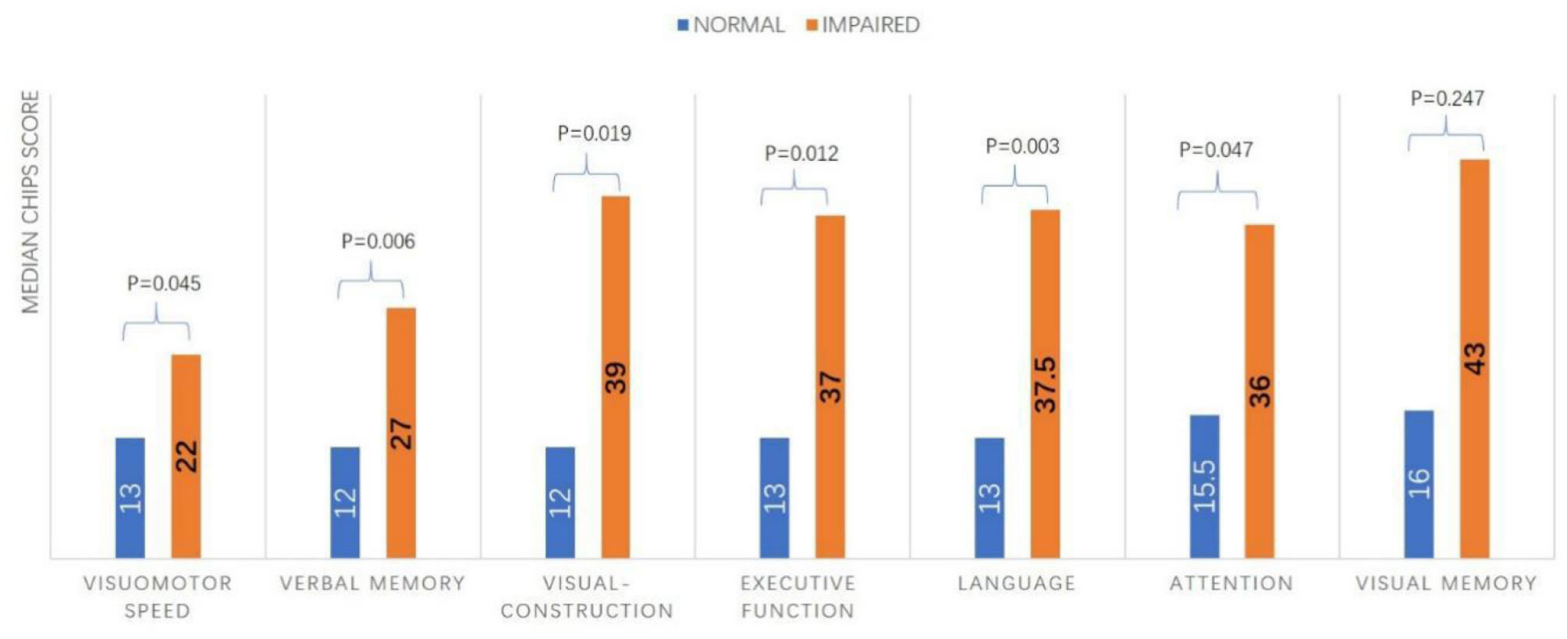
Comparison of the CHIPS score according to impairment in each cognitive domain.

Of the 103 patients who completed the modified VDB assessment at 3 months and according to the modified VDB assessment, 48 patients had no impaired cognitive domain. The distribution of numbers of impaired cognitive domains were: one (20 patients), two (18 patients), three (seven patients), four (six patients), five (one patient), six (three patients), and all seven domains (one patient). The most commonly impaired cognitive domain was visuomotor speed with impairment found in 28 (27.2%) cases, followed by verbal memory impairment in 26 (25.2%) cases. Visual memory was least commonly impaired with only nine (8.7%) cases exhibiting visual memory impairment (Supplementary Figure 1). By definition, a total of 55 patients had PSCI, of which 43 (41.7%) were PSCI-ND and 12 (11.7%) were PSD patients. The comparisons of CHIPS scores in terms of each cognitive domain impairment or not are shown in Supplementary Table 4. It reveals that, except for the visual memory, CHIPS scores were significantly higher in patients with impairment in all other cognitive domains (Figure 3).

### Univariate Analysis of PSCI

The univariate analysis reveals that patients with PSCI were older, had higher IQCODE scores, had more frequent previous stroke history and atrial fibrillation, had higher CHIPS scores, and had more severe WMLs and MTLA. Additionally, patients with PSCI had higher HAMD scores at 3 months (Table 1). Furthermore, PSCI patients had severer impairment in each zone of the CCP in comparison to those with non-PSCI (Supplementary Table 2).

### Multivariate Logistic Regression Analysis of PSCI

The independent variables with values of *P* < 0.05 in the univariate analysis results (Table 1) were subjected to Spearman's correlation analysis to check the colinearity, including age, IQCODE score, ischemic stroke history, atrial fibrillation history, infratentorial acute infarction, total CHIPS score, PVH, DWMH, MTLA, and HAMD. Age was significantly correlated with IQCODE score, PVH score, and MTLA score (correlation coefficient *r* = 0.566, 0.509, and 0.549, respectively). Additionally, PVH score was significantly correlated with DWMH score, CHIPS score, and MTLA score (*r* = 0.675, 0.586, and 0.557, respectively), and CHIPS score was also significantly correlated with DWMH score and MTLA score (*r* = 0.713 and 0.529, respectively). There were no significant correlations between any other independent variables (the correlation coefficients were all <0.5). Because both age and IQCODE score are important factors affecting PSCI, we set up two models to analyze them. Model 1 included age, and model 2 included IQCODE score with the rest of the factors held constant; history of ischemic stroke, history of atrial fibrillation, acute infratentorial infarction, CHIPS score, and HAMD score served as independent variables.

The logistic regression of model 1 revealed that age, CHIPS score, and HAMD score were independent risk factors for PSCI at 3 months, whereas model 2 revealed that IQCODE, CHIPS, and HAMD scores were independent risk factors for PSCI at 3 months (Table 2). Compared with model 2, the predictive probability of model 1 was greater (80.6 vs. 74.8%), and the model fit was higher (Cox and Snell *R*^2^ = 0.377 vs. 0.309; Nagelkerke *R*^2^ = 0.504 vs. 0.413).

**Table 2:** Multivariate logistic regression of risk factors for PSCI.

		***B***	***OR* (95% CI)**	***P-*value**
Model 1	Age (years)	0.078	1.081 (1.032–1.132)	0.001
	Ischemic stroke history	−0.117	0.890 (0.178–4.443)	0.887
	Atrial fibrillation	1.570	4.805 (0.540–42.735)	0.159
	Acute infratentorial infarction	−0.844	0.430 (0.141–1.313)	0.138
	Total CHIPS score	0.034	1.034 (1.006–1.063)	0.016
	HAMD	0.148	1.160 (1.047–1.284)	0.004
Model 2	IQCODE score	1.405	4.074(2.064–8.041)	<0.001
	Ischemic stroke history	−0.151	0.860 (0.160–4.615)	0.860
	Atrial fibrillation	1.417	4.378 (0.445–43.025)	0.205
	Acute infratentorial infarction	−0.437	0.646 (0.199–2.094)	0.467
	Total CHIPS score	0.037	1.038 (1.009–1.067)	0.009
	HAMD	0.115	1.122 (1.011–1.246)	0.030

*OR, odds ratio; CI, confidence interval; CHIPS, Cholinergic Pathways Hyperintensities Scale; HAMD, Hamilton Depression Rating Scale; IQCODE, Informant Questionnaire on Cognitive Decline in the Elderly*.

As the history of stroke was an important risk factor for PSCI, we conducted another regression model in patients without stroke history (Supplementary Table 3). The analysis revealed that, despite adjusting for the previous stroke, higher CHIPS scores were also associated with PSCI at 3 months.

Ordinal regression analysis revealed that more severe cognitive dysfunction was significantly associated with higher mRS, adjusted for age, education level, IQCODE, NIHSS score at admission, and PVH (Table 3).

**Table 3:** Ordinal regression analysis of mRS at 3 months^*^.

	**Estimate**	**95% C.I**.	***P-*value**
Age	−0.010	−0.051–0.031	0.636
IQCODE score	0.133	−0.376–0.643	0.608
Education level	0.387	−0.217–0.990	0.209
NIHSS score at admission	0.165	0.047–0.284	0.006
PVH	0.301	−0.164–0.767	0.204
Cognitive status	1.176	0.431–1.920	0.002

*IQCODE, Informant Questionnaire on Cognitive Decline in the Elderly; NIHSS, National Institutes of Health Stroke Scale; PVH, periventricular hyperintensity*.

* *Cognitive status includes normal cognitive function, PSCI-ND and PSD*.

## Discussion

In this longitudinal observational study, we assessed the associations between PSCI and lesions involving the CCP in Chinese patients with early subacute ischemic stroke. Our main finding was that a higher CHIPS score was associated with the presence of PSCI at 3 months after stroke adjusted for possible confounders, indicating CCP lesions might play a role in PSCI.

In the present study, the incidence of PSCI at 3 months after a mild-to-moderate index stroke was 53.4%. This finding was similar to the results of several previous studies (2, 39). Differences in the reported incidence of PSCI may be attributed to different definitions of PSCI, use of different assessment tools, different sample sizes, the healthcare institutions in which the patients were treated, and different therapeutic management.

Our findings suggest that severe lesions involving the CCP may be associated with cognitive impairment at 3 months after an ischemic stroke. The reduction of certain neural matrices in the cholinergic pathway or the disruption of information transmission on this pathway may lead to a disruption of the neural network connections, potentially contributing to newly developed PSD (17). The lesions measured in CCP using the CHIPS included WMH lesions on T2-weighted MRI, which might have been caused by chronic and acute vascular damage. All these lesions involving the CCP may affect information transmission in the central acetylcholinergic system, thereby affecting cognitive and modulate attentional functions. Additionally, some studies suggest that the pathogenesis of PSCI may include the coexistence of neurodegenerative pathology and cerebrovascular damage in patients after stroke, which mutually promote and may contribute to cognitive impairment (11, 39–41). Furthermore, several studies demonstrate that PSCI development and a poor functional prognosis after stroke are not only the result of acute ischemic stroke lesions; the adaptive capacity of the brain (including brain reserve and cognitive reserve capacities) before stroke also plays a vital role (42). CHIPS not only measures lesions on the CCP caused by old-/new-onset infarction, but also by aging or disease-related changes in the white matter leading to a disruption of cholinergic fibers by strategically located WMHs (20). It is, therefore, reasonable to take new-onset acute infarction, old infarction, and WMHs into consideration when calculating the CHIPS score.

As previously reported in other studies, we found that age and IQCODE score were important risk factors for PSCI (43, 44). Previous studies indirectly suggest that PSCI is closely related to brain cognitive function reserve before stroke. The present results indicate that higher depressive scores may be associated with PSCI. Several other studies reported similar results, depressive status was closely associated with cognitive impairment in stroke patients (1, 41) because lesion damage can induce inflammation, and circulating cytokines and immune responses may promote depression and cognitive impairment (45).

Our study did not distinguish the impact of left and right hemisphere damage on cognitive function or CCP. As required by most studies, the VDB test requires that the patient does not have significant aphasia. Therefore, patients with severe language impairment most likely caused by left-hemispheric infarction had been excluded, resulting in a potentially selected bias. Previous research also shows that the transmission of cholinergic signals in the brain is network transmission not just limited to the unilateral cerebral hemisphere (15). Thus, it is not reliable to assess the impact of left or right hemisphere lesions on CCP and cognitive outcomes.

According to Table 2, the current results indicate that severe lesions of CCP may be correlated with PSCI in patients with recurrent or first-ever stroke. Many previous studies show that people with multiple strokes are substantially more likely to have PSCI than people with the first stroke. According to Supplementary Table 3, involving a regression model that did not include patients with previous stroke also suggest that higher CHIPS scores were associated with PSCI. These results indicate that CCP is a major risk factor for PSCI not only in patients with first-ever stroke but also those with multiple strokes.

The current findings also reveal that PSCI was associated with more severe disability at 3 months, which suggests that it is important to screen for and treat PSCI after stroke. However, in the current study, the NIHSS scores of the included patients were lower, and most patients had mild mRS scores at 3 months after stroke, potentially generating a statistical offset.

As a strength of the present study, we collected relatively detailed clinical, imaging, and follow-up data from early subacute ischemic stroke patients using a prospective research method. The second strength was the use of comprehensive MRI assessments, including acute cerebral infarction assessment and preexisting abnormalities assessments, which include CMBs, MTLA, and WMLs. Third, when the promotion of advanced MRI is limited, we can use the CHIPS score, the semiquantitative MR score, to assess the degree of CCP damage, which has been rarely investigated in acute ischemic stroke. However, this study was also characterized by several limitations: (1) The rate of loss to follow-up in the present study was relatively high, and the sample size was relatively small. (2) The IQCODE is a global assessment of cognitive impairment prestroke; it is inadequate for rigorous assessment of specific cognitive domains. Therefore, residual confounding of prestroke cognitive function remains possible. (3) Although we have excluded obvious visual and auditory impairments, neurological diseases and previous psychiatric history based on medical records and physical examinations, we did not conduct a detailed evaluation of these conditions, which might affect the results of the cognitive assessment. (4)NIHSS, mRS, and ADL scores at 3 months for most patients were low, and patients with severe stroke were excluded, potentially limiting the generalizability of the current findings. (5) MRI with a slice thickness/gap of 6 mm/1 mm cannot accurately measure the degree of CCP lesions, which means that we may have missed small lesions in the CCP. (6) We only used semiquantitative measurement tools to assess MRI features. These tools cannot accurately distinguish and measure cholinergic nerve pathways and other related nerve pathway fibers (e.g., glutaminergic and other neurotransmitter pathways), nor can they evaluate certain measurements that may predict cognitive function, such as cortical thickness or hippocampal volume.

In conclusion, the incidence of PSCI in the present study population was 53.4%. The severity of lesions involving the CCP may be associated with cognitive impairment at 3 months after stroke. Additionally, cognitive function status is closely related to prognostic outcome and can affect stroke survivors' quality of life.

## Data Availability Statement

The original contributions presented in the study are included in the article/Supplementary Material, further inquiries can be directed to the corresponding author/s.

## Ethics Statement

The study protocol was approved by the Ethics Committee of Dongguan People's Hospital (no. KYKT2018-006).

## Author Contributions

J-fQ, H-HZ, W-MX, and Y-kC are the principal investigator that designed the study, carried out the quantitative analysis, and drafted the article. Y-lL and W-cL collected the data. Z-qW and D-hQ reviewed and edited the manuscript. All authors have read and approved the final manuscript.

## Conflict of Interest

The authors declare that the research was conducted in the absence of any commercial or financial relationships that could be construed as a potential conflict of interest.

## References

[1] Jokinen H Melkas S Ylikoski R Pohjasvaara T Kaste M Erkinjuntti T. Post-stroke cognitive impairment is common even after successful clinical recovery. Eur J Neurol. (2015) 22:1288–94. 10.1111/ene.12743 26040251

[2] Barbay M Taillia H Nedelec-Ciceri C Bompaire F Bonnin C Varvat J. Prevalence of poststroke neurocognitive disorders using national institute of neurological disorders and stroke-Canadian Stroke Network, VASCOG Criteria (Vascular Behavioral and Cognitive Disorders), and optimized criteria of cognitive deficit. Stroke. (2018) 49:1141–7. 10.1161/STROKEAHA.117.018889 29643258

[3] Oksala NK Jokinen H Melkas S Oksala A Pohjasvaara T Hietanen M. Cognitive impairment predicts poststroke death in long-term follow-up. J Neurol Neurosurg Psychiatry. (2009) 80:1230–5. 10.1136/jnnp.2009.174573 19620138

[4] Lee M Saver JL Hong KS Wu YL Liu HC Rao NM. Cognitive impairment and risk of future stroke: a systematic review and meta-analysis. Cmaj. (2014) 186:536–46. 10.1503/cmaj.140147 25157064 PMC4188684

[5] Francis PT. Glutamatergic approaches to the treatment of cognitive and behavioural symptoms of Alzheimer's disease. Neurodegener Dis. (2008) 5:241–3. 10.1159/000113713 18322401

[6] Lamour Y Dutar P Rascol O Jobert A. Basal forebrain neurons projecting to the rat frontoparietal cortex: electrophysiological and pharmacological properties. Brain Res. (1986) 362:122–31. 10.1016/0006-8993(86)91405-8 3002548

[7] Wenk GL. Pharmacological manipulations of the substantia innominata-cortical cholinergic pathway. Neurosci Lett. (1984) 51:99–103. 10.1016/0304-3940(84)90269-6 6151154

[8] Kurosawa M Sato A Sato Y. Stimulation of the nucleus basalis of Meynert increases acetylcholine release in the cerebral cortex in rats. Neurosci Lett. (1989) 98:45–50. 10.1016/0304-3940(89)90371-6 2565563

[9] Brown DA. Muscarinic acetylcholine receptors (mAChRs) in the nervous system: some functions and mechanisms. J Mol Neurosci. (2010) 41:340–6. 10.1007/s12031-010-9377-2 20446119

[10] Dajas-Bailador F Wonnacott S. Nicotinic acetylcholine receptors and the regulation of neuronal signalling. Trends Pharmacol Sci. (2004) 25:317–24. 10.1016/j.tips.2004.04.006 15165747

[11] Selden NR Gitelman DR Salamon-Murayama N Parrish TB Mesulam MM. Trajectories of cholinergic pathways within the cerebral hemispheres of the human brain. Brain. (1998) 121:2249–57. 10.1093/brain/121.12.2249 9874478

[12] Mesulam MM. The cholinergic innervation of the human cerebral cortex. Prog Brain Res. (2004) 145:67–78. 10.1016/S0079-6123(03)45004-8 14650907

[13] Riascos D Nicholas A Samaeekia R Yukhananov R Mesulam MM Bigio EH. Alterations of Ca^2+^-responsive proteins within cholinergic neurons in aging and Alzheimer's disease. Neurobiol Aging. (2014) 35:1325–33. 10.1016/j.neurobiolaging.2013.12.017 24461366 PMC3961506

[14] Bloem B Schoppink L Rotaru DC Faiz A Hendriks P Mansvelder HD. Topographic mapping between basal forebrain cholinergic neurons and the medial prefrontal cortex in mice. J Neurosci. (2014) 34:16234–46. 10.1523/JNEUROSCI.3011-14.2014 25471564 PMC6608490

[15] Chandler D Waterhouse BD. Evidence for broad versus segregated projections from cholinergic and noradrenergic nuclei to functionally and anatomically discrete subregions of prefrontal cortex. Front Behav Neurosci. (2012) 6:20. 10.3389/fnbeh.2012.00020 22661934 PMC3356860

[16] Bocti C Swartz RH Gao FQ Sahlas DJ Behl P Black SE. A new visual rating scale to assess strategic white matter hyperintensities within cholinergic pathways in dementia. Stroke. (2005) 36:2126–31. 10.1161/01.STR.0000183615.07936.b6 16179569

[17] Lim JS Kim N Jang MU Han MK Kim S Baek MJ. Cortical hubs and subcortical cholinergic pathways as neural substrates of poststroke dementia. Stroke. (2014) 45:1069–76. 10.1161/STROKEAHA.113.004156 24603067

[18] Furie KL Jayaraman MV. 2018 guidelines for the early management of patients with acute ischemic stroke. Stroke. (2018) 49:509–10. 10.1161/STROKEAHA.118.020176 29367335

[19] Harrison JK Fearon P Noel-Storr AH McShane R Stott DJ Quinn TJ. Informant Questionnaire on Cognitive Decline in the Elderly (IQCODE) for the diagnosis of dementia within a secondary care setting. Cochrane Database Syst Rev. (2015) 3:CD010772. 10.1002/14651858.CD010772.pub2 25754745

[20] Qu JF Chen YK Luo GP Zhao JH Zhong HH Yin HP. Severe lesions involving cortical cholinergic pathways predict poorer functional outcome in acute ischemic stroke. Stroke. (2018) 49:2983–9. 10.1161/STROKEAHA.118.023196 30571427 PMC6257508

[21] Fazekas F Chawluk JB Alavi A Hurtig HI Zimmerman RA. MR signal abnormalities at 1.5 T in Alzheimer's dementia and normal aging. Am J Roentgenol. (1987) 149:351–6. 10.2214/ajr.149.2.351 3496763

[22] Scheltens P Leys D Barkhof F Huglo D Weinstein HC Vermersch P. Atrophy of medial temporal lobes on MRI in “probable” Alzheimer's disease and normal ageing: diagnostic value and neuropsychological correlates. J Neurol Neurosurg Psychiatry. (1992) 55:967–72. 10.1136/jnnp.55.10.967 1431963 PMC1015202

[23] Pendlebury ST Klaus SP Thomson RJ Mehta Z Wharton RM Rothwell PM. Methodological factors in determining risk of dementia after transient ischemic attack and stroke: (III) applicability of cognitive tests. Stroke. (2015) 46:3067–73. 10.1161/STROKEAHA.115.010290 26463688 PMC5321486

[24] Mijajlović MD Pavlović A Brainin M Heiss WD Quinn TJ Ihle-Hansen HB. Post-stroke dementia - a comprehensive review. BMC Med. (2017) 15:11. 10.1186/s12916-017-0779-7 28095900 PMC5241961

[25] Nasreddine ZS Phillips NA Bédirian V Charbonneau S Whitehead V Collin I. The Montreal Cognitive Assessment, MoCA: a brief screening tool for mild cognitive impairment. J Am Geriatr Soc. (2005) 53:695–9. 10.1111/j.1532-5415.2005.53221.x 15817019

[26] Godefroy O Yaïche H Taillia H Bompaire F Nédélec-Ciceri C Bonnin C. Who should undergo a comprehensive cognitive assessment after a stroke? A cognitive risk score. Neurology. (2018) 91:e1979–87. 10.1212/WNL.0000000000006544 30333160 PMC6260202

[27] Tang WK Chen YK Lu JY Wong A Mok V Chu WC. Absence of cerebral microbleeds predicts reversion of vascular 'cognitive impairment no dementia' in stroke. Int J Stroke. (2011) 6:498–505. 10.1111/j.1747-4949.2011.00682.x 22111793

[28] Tham W Auchus AP Thong M Goh ML Chang HM Wong MC. Progression of cognitive impairment after stroke: 1year results from a longitudinal study of Singaporean stroke patients. J Neurol Sci. (2002) 203–204:49–52. 10.1016/S0022-510X(02)00260-5 12417356

[29] Mok VC Wong A Yim P Fu M Lam WW Hui AC. The validity and reliability of Chinese frontal assessment battery in evaluating executive dysfunction among Chinese patients with small subcortical infarct. Alzheimer Dis Assoc Disord. (2004) 18:68–74. 10.1097/01.wad.0000126617.54783.7 15249850

[30] Royall DR. The FAB: a frontal assessment battery at bedside. Neurology. (2001) 57:565. 10.1212/WNL.57.3.565 11502945

[31] Wechsler D. Wechsler Adult Intelligence Scale, 3rd version. New York, NY: The Psychological Corporation (1997). 10.1037/t49755-000

[32] Wechsler D. Wechsler Memory Scale, 3rd version. San Antonio: The Psychological Corporation (1997).

[33] Cheung RW Cheung MC Chan AS. Confrontation naming in Chinese patients with left, right or bilateral brain damage. J Int Neuropsychol Soc. (2004) 10:46–53. 10.1017/S1355617704101069 14751006

[34] Chiu HF Chan CK Lam LC Ng KO Li SW Wong M. The modified Fuld Verbal Fluency Test: a validation study in Hong Kong. J Gerontol B Psychol Sci Soc Sci. (1997) 52:P247–50. 10.1093/geronb/52B.5.P247 9310094

[35] Sunderland T Hill JL Mellow AM Lawlor BA Gundersheimer J Newhouse PA. Clock drawing in Alzheimer's disease. A novel measure of dementia severity. J Am Geriatr Soc. (1989) 37:725–9. 10.1111/j.1532-5415.1989.tb02233.x 2754157

[36] Smith A. Symbol Digit Modalities Test. Los Angeles: Western Psychological Services (1991).

[37] American Psychiatric Association. Diagnostic and Statistical Manual of Mental Disorders. Arlington, VA: American Psychiatric Publishing (2013). 10.1176/appi.books.9780890425596

[38] Lawton MP Brody EM. Assessment of older people: self-maintaining and instrumental activities of daily living. Gerontologist. (1969) 9:179–86. 10.1093/geront/9.3_Part_1.179 5349366

[39] Picciotto MR Higley MJ Mineur YS. Acetylcholine as a neuromodulator: cholinergic signaling shapes nervous system function and behavior. Neuron. (2012) 76:116–29. 10.1016/j.neuron.2012.08.036 23040810 PMC3466476

[40] Micheau J Marighetto A. Acetylcholine and memory: a long, complex and chaotic but still living relationship. Behav Brain Res. (2011) 221:424–9. 10.1016/j.bbr.2010.11.052 21130809

[41] Hasselmo ME Sarter M. Modes and models of forebrain cholinergic neuromodulation of cognition. Neuropsychopharmacology. (2011) 36:52–73. 10.1038/npp.2010.104 20668433 PMC2992803

[42] Mok VC Lam BY Wong A Ko H Markus HS Wong LK. Early-onset and delayed-onset poststroke dementia - revisiting the mechanisms. Nat Rev Neurol. (2017) 13:148–59. 10.1038/nrneurol.2017.16 28211452

[43] Akinyemi RO Allan L Owolabi MO Akinyemi JO Ogbole G Ajani A. Profile and determinants of vascular cognitive impairment in African stroke survivors: the CogFAST Nigeria Study. J Neurol Sci. (2014) 346:241–9. 10.1016/j.jns.2014.08.042 25238666

[44] Kovalenko EA Bogolepova AN Katunin DA. The role of pre-stroke cognitive disorders in the formation of post-stroke cognitive impairment. Zh Nevrol Psikhiatr Im S S Korsakova. (2017) 117:19–24. 10.17116/jnevro201711712219-24 29411741

[45] Pessoa Rocha N Reis HJ Vanden Berghe P Cirillo C. Depression and cognitive impairment in Parkinson's disease: a role for inflammation and immunomodulation? Neuroimmunomodulation. (2014) 21:88–94. 10.1159/000356531 24557040

