# Supplementary material for: Severity of Lesions Involving the Cortical Cholinergic Pathways May Be Associated With Cognitive Impairment in Subacute Ischemic Stroke
Source: Front Neurol. 2021 Jun 8;12:606897. doi: 10.3389/fneur.2021.606897 (PMC8217623; doi:10.3389/fneur.2021.606897)
Supplement: Supplementary file 1 [file Data_Sheet_1.zip › Supplemental_Material/Supplemental Table 1.docx]

**Supplemental Table 1** Neuropsychological test battery for the modified version of the Vascular Dementia Battery.

| Domains | Neuropsychological tests |
| --- | --- |
| Executive function | Frontal Assessment Battery (FAB) |
|  | Trail making test, parts A and B |
| Attention | Digit span (forward and backward) |
|  | Auditory detection test |
| Language | Modified Boston Naming Test |
|  | Verbal fluency: animal |
|  | Verbal fluency: vegetables |
| Verbal memory | Immediate and delayed word list recall |
|  | Delayed word list recognition |
|  | Immediate and delayed story recall |
| Visual memory | Picture recall |
|  | Immediate recall |
|  | Delayed recall |
| Visual-construction | Clock-drawing test |
|  | Visual reproduction (copy) |
| Visuomotor speed | Symbol Digit Modalities Test |
|  | Digit cancellation test |
|  | Maze task |
