# Supplementary material for: Severity of Lesions Involving the Cortical Cholinergic Pathways May Be Associated With Cognitive Impairment in Subacute Ischemic Stroke
Source: Front Neurol. 2021 Jun 8;12:606897. doi: 10.3389/fneur.2021.606897 (PMC8217623; doi:10.3389/fneur.2021.606897)
Supplement: Supplementary file 1 [file Data_Sheet_1.zip › Supplemental_Material/Supplemental Table 2.docx]

**Supplemental Table 2** Comparison of the four levels of CHIPS scores between patients with normal cognition and patients with PSCI.

|  | Normal  N=48 | PSCI  N=55 | z-value | *P-value* |
| --- | --- | --- | --- | --- |
| low external capsule layer | 4（0-8） | 12（3-16） | -3.004 | 0.003 |
| high external capsule layer | 3（0-6） | 9（2-12） | -3.480 | 0.001 |
| corona radiata | 2（0-3.75） | 6（2-8） | -4.235 | ＜0.001 |
| semioval center | 0（0-2） | 2（0-5） | -3.284 | 0.001 |
