# Supplementary material for: Severity of Lesions Involving the Cortical Cholinergic Pathways May Be Associated With Cognitive Impairment in Subacute Ischemic Stroke
Source: Front Neurol. 2021 Jun 8;12:606897. doi: 10.3389/fneur.2021.606897 (PMC8217623; doi:10.3389/fneur.2021.606897)
Supplement: Supplementary file 1 [file Data_Sheet_1.zip › Supplemental_Material/Supplemental Table 3.docx]

**Supplemental Table 3** Multivariate logistic regression of risk factors for PSCI in patients without a history of stroke.

|  |  | *Β* | *OR (95% CI)* | *P-*value |
| --- | --- | --- | --- | --- |
| Model 1 | Age (years) | 0.076 | 1.079 (1.025-1.135) | 0.003 |
|  | Education levle | -0.567 | 0.567 (0.219-1.469) | 0.243 |
|  | Atrial fibrillation | 1.339 | 3.815 (0.364-39.954) | 0.264 |
|  | Acute infratentorial infarction | −0.817 | 0.442 (0.129-1.515) | 0.194 |
|  | MTLA | 0.050 | 1.051(0.739-1.496) | 0.782 |
|  | Total CHIPS score | 0.041 | 1.042 (1.009-1.075) | 0.013 |
|  | HAMD | 0.167 | 1.182 (1.058-1.320) | 0.003 |
|  |  |  |  |  |
| Model 2 | IQCODE score | 1.400 | 4.056(1.878-8.755) | ＜0.001 |
|  | Education levle | -0.555 | 0.574 (0.234-1.406) | 0.224 |
|  | Atrial fibrillation | 1.408 | 4.090 (0.349-47.889) | 0.262 |
|  | Acute infratentorial infarction | -0.250 | 0.779 (0.211-2.880) | 0.708 |
|  | MTLA | -0.011 | 0.989 (0.685-1.429) | 0.954 |
|  | Total CHIPS score | 0.046 | 1.047 (1.013-1.082) | 0.006 |
|  | HAMD | 0.129 | 1.137 (1.014-1.275) | 0.028 |

**Abbreviations:** OR, odds ratio; CI, confidence interval; MTLA, medial temporal lobe atrophy; CHIPS, Cholinergic Pathways Hyperintensities Scale; HAMD, Hamilton Depression Rating Scale; IQCODE, Informant Questionnaire on Cognitive Decline in the Elderly.

Note: Compared with model 1, model 2 had greater predictive probability (model 2: 81.8% vs. model 1: 74.2%), and had a better model fit (Cox and Snell *R^2^* = 0.409 vs. 0.362; Nagelkerke *R^2^* = 0.545 vs. 0.483).
