# Supplementary material for: Severity of Lesions Involving the Cortical Cholinergic Pathways May Be Associated With Cognitive Impairment in Subacute Ischemic Stroke
Source: Front Neurol. 2021 Jun 8;12:606897. doi: 10.3389/fneur.2021.606897 (PMC8217623; doi:10.3389/fneur.2021.606897)
Supplement: Supplementary file 1 [file Data_Sheet_1.zip › Supplemental_Material/Supplemental Table 4.docx]

**Supplemental Table 4** The comparison of CHIPS scores according to each impaired cognitive domain.

| *P-*value | CHIPS score |  |  |
| --- | --- | --- | --- |
| 0.045 | 13(4-32) | N | Visuomotor speed |
|  | 22(8-46.5) | Imp |  |
| 0.019 | 12(3-34) | N | Verbal memory |
|  | 27(10.5-47.5) | Imp |  |
| 0.006 | 12(3.5-29) | N | Visual-construction |
|  | 39(15-55.5) | Imp |  |
| 0.012 | 13(4-31.5) | N | Executive function |
|  | 37(15-53.5) | Imp |  |
| 0.003 | 13(3.5-31.5) | N | Language |
|  | 37.5(23-48.5) | Imp |  |
| 0.047 | 15(4-32.5) | N | Attention |
|  | 36(7-53) | Imp |  |
| 0.247 | 16(4-35) | N | Visual memory |
|  | 43(3.5-47) | Imp |  |

**Abbreviations:** CHIPS, Cholinergic Pathways Hyperintensities Scale.

**Note:** N= Nomal; Imp= Impairment.
