# Supplementary figures and images for: Severity of Lesions Involving the Cortical Cholinergic Pathways May Be Associated With Cognitive Impairment in Subacute Ischemic Stroke
Source: Front Neurol. 2021 Jun 8;12:606897. doi: 10.3389/fneur.2021.606897 (PMC8217623; doi:10.3389/fneur.2021.606897)

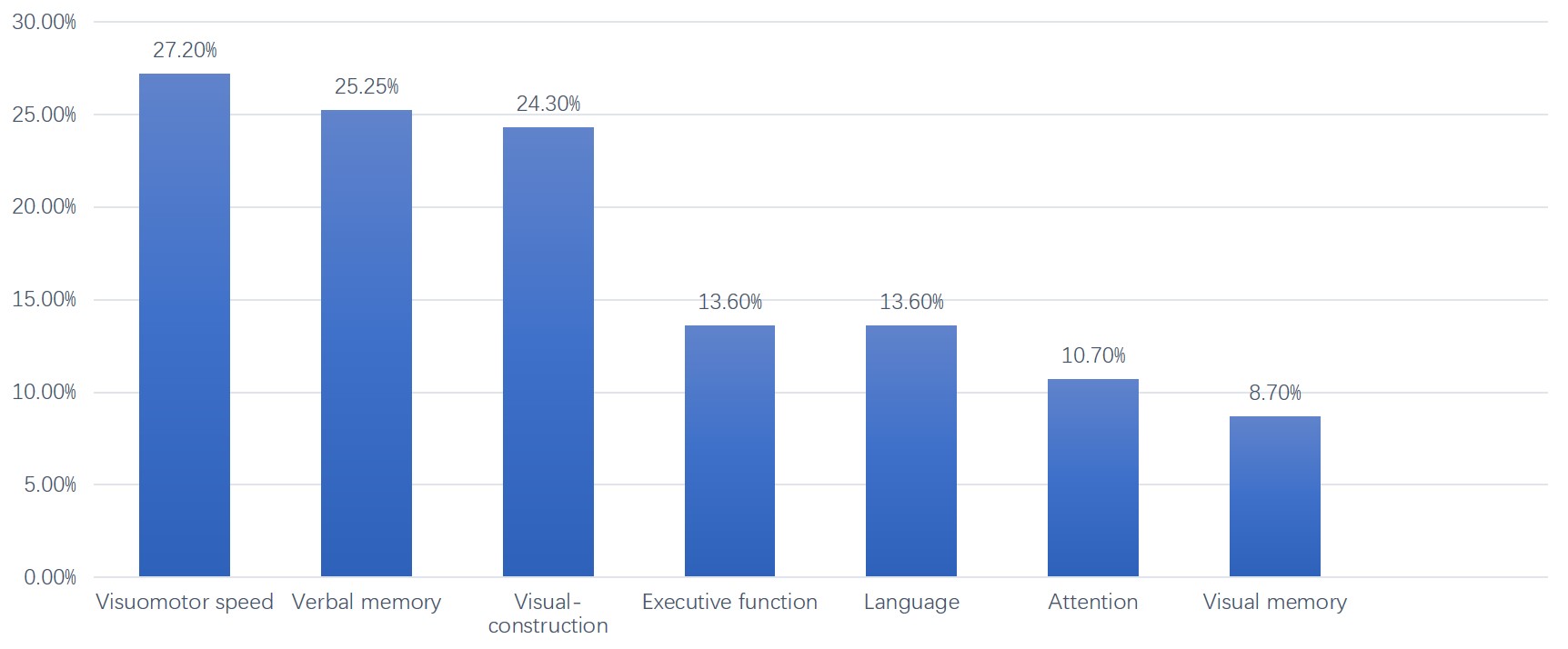

Supplement: Supplementary file 1 [file Data_Sheet_1.zip › Supplemental_Material/Supplemental Figure 1.jpg]
